# Efficacy of Olanzapine for High and Moderate Emetogenic Chemotherapy in Children

**DOI:** 10.3390/children7090140

**Published:** 2020-09-16

**Authors:** So Rae Lee, Su Min Kim, Min Young Oh, Jae Min Lee

**Affiliations:** 1 Department of Medicine, College of Medicine, Yeungnam University, Daegu 42415, Korea; dlthfo@naver.com (S.R.L.); gogo_sumin@naver.com (S.M.K.); polarbear_yn@naver.com (M.Y.O.); 2 Department of Pediatrics, College of Medicine, Yeungnam University, Daegu 42415, Korea

**Keywords:** adolescent, antiemetics, childhood cancer, chemotherapy-induced nausea and vomiting, olanzapine

## Abstract

This study was conducted to investigate the safety and efficacy of olanzapine for high and moderate emetogenic chemotherapy in children and young adults. We retrospectively reviewed the records of pediatric patients (*n* = 13) with cancer who had been administered olanzapine as an anti-emetic drug (AED) during a high and moderate emetogenic chemotherapy block from January 2018 to March 2020. Patients were administered other prophylactic AEDs according to practice guidelines. The mean age of the patients was 14.1 ± 5.5 years. The total number of chemotherapy cycles was 41. Twenty-one (51.2%) chemotherapy blocks were high emetogenic chemotherapy and 20 (48.8%) blocks were moderate emetogenic chemotherapy. Olanzapine was used for prophylaxis in 20 (48.8%) blocks of chemotherapy and rescue in 21 (51.2%). Of the 41 cycles, a complete response to olanzapine was achieved in 31 (75.6%), partial response in 6 (14.6%), and no response in 4 (9.8%). The mean dose was 0.07 ± 0.04 mg/kg/dose and 2.50 ± 1.37 mg/m^2^/dose. Adverse effects included somnolence, hyperglycemia, fatigue, and disturbed sleep. Our findings indicate that olanzapine was effective and safe for treating chemotherapy-induced nausea and vomiting in children. A prospective controlled study is needed to confirm these findings.

## 1. Introduction

Chemotherapy-induced nausea and vomiting (CINV) is a frequent and potentially treatment-limiting complication of cancer therapy in both adults and children [1]. Despite the appropriate use of prophylactic antiemetics, breakthrough and refractory CINV often occurs, for which there is no effective treatment in children [2,3].

Olanzapine is well-known as an antipsychotic drug used to treat schizophrenia and depression in children and adolescents [4,5,6,7]. It has long been used in children because of its safety [8]. Olanzapine exerts antiemetic effects by acting on various neurotransmitters that cause vomiting and nausea [4]. Recent studies have investigated the use of olanzapine as an antiemetic drug (AED) in adult patients undergoing chemotherapy. The findings revealed an effective antiemetic effect compared with aprepitant or metoclopramide and other combinations of AEDs [9,10,11,12,13,14,15].

However, few studies have focused on olanzapine use in children [2,3,16]. In Korea, the Korea Food and Drug Administration permits olanzapine administration only for pediatric psychiatric diseases; therefore, there is no reported evidence of its use as an AED in pediatric patients with cancer.

The purpose of this study was to investigate the efficacy and safety of off-label use of olanzapine as an AED in children and young adults with cancer being administered moderate and high emetogenic chemotherapy.

## 2. Materials and Methods

### 2.1. Patients

Data of children who were administered moderate and high emetogenic chemotherapy and olanzapine for CIV control at Yeungnam University Hospital from January 2018 to March 2020 were retrospectively analyzed. We reviewed the patients’ medical, nursing, medication, and chemotherapy records, laboratory results, and vital charts. To investigate the adverse effects of olanzapine, we reviewed the nursing records for symptoms of pain, fatigue, somnolence, disturbed sleep, lack of appetite, dry mouth, or mood changes.

### 2.2. Chemotherapy Emetogenicity Assessment

The emetogenicity of chemotherapy drugs was classified according to the 2019 guideline for the classification of the acute emetogenic potential of antineoplastic medication in pediatric patients with cancer [17]. The emetogenic potential of anticancer drugs that are not described in this guideline was determined according to the 2011 guidelines [18].

### 2.3. Standard CIV Prophylaxis

Antiemetics practice was performed according to the POGO guidelines [19].

For blocks administered high emetogenic chemotherapy (HEC), dexamethasone (if corticosteroids were permitted), aprepitant (if ≥12 years old) (Emend, MSD, Whitehouse Station, NJ, USA), and/or 5-HT3 antagonists (palonosetron; Aloxi, Helsinn Birex Pharmaceuticals Ltd., Dublin, Ireland) were used for CIV prophylaxis.

For blocks administered moderate emetogenic chemotherapy (MEC), dexamethasone (if corticosteroids were permitted) and/or 5-HT3 antagonists (palonosetron; Aloxi, Helsinn Birex Pharmaceuticals Ltd.Dublin, Ireland) were used for CIV prophylaxis.

### 2.4. Olanzapine Treatment

Olanzapine was administered for two different purposes: prophylaxis and rescue. First, it was administered for rescue; for patients in whom olanzapine was effective in rescue therapy, olanzapine prophylaxis was added from the next day or next chemotherapy block. For prophylaxis, olanzapine was orally administered 1 h prior to chemotherapy with or without other AEDs. In the rescue group, dexamethasone, 5-HT3 receptor antagonists, and aprepitant were used as prophylactic AEDs, but additional olanzapine was ultimately required for uncontrolled vomiting. When vomiting was not controlled by olanzapine, additional AEDs, such as metoclopramide, palonosetron, ondansetron, or diazepam were used. Olanzapine was initially administered orally at 0.1–0.14 mg/kg/dose (maximum 10 mg/dose) once daily. If there were no side effects, but nausea and vomiting occurred at night, olanzapine was administered twice daily. Dose modification was performed in 1.25 mg increments while monitoring for antiemetic and adverse effects. For olanzapine, Zyprexa (2.5 mg, 5 mg, and 10 mg; Lilly, Indianapolis, IN, USA) and Zyprexa zydis (5 mg and 10 mg; Lilly, Indianapolis, IN, USA) were used. Olanzapine was used as an antiemetic following approval for off-label drug use from the Health Insurance Review and Assessment Service and after receiving institutional review board approval.

### 2.5. Response Assessment

The CIV response of olanzapine was retrospectively assessed by reviewing the nursing and medication records. We counted the number of vomiting episodes in the nursing records and checked the number of additional AEDs used throughout the acute CIV phase (within 24 h after chemotherapy) in the medication records. We defined a complete response (CR) as no vomiting or additional AEDs recorded in the medical records throughout the acute phase. A partial response (PR) was defined as either vomiting more than once without additional AED use or additional AED use without vomiting. No response (NR) was defined as additional AED use and >1 episode of vomiting. Adverse events were graded according to National Cancer Institute Common Toxicity Criteria (version 4) [20].

### 2.6. Ethical Approval

This study was approved by the institutional review board of Yeungnam University Hospital (IRB No. YUMC 2019-08-014). All procedures performed in studies involving human participants were conducted in accordance with the ethical standards of the institutional research committee (Yeungnam University Hospital; IRB No. YUMC 2020-09-017).

## 3. Results

### 3.1. Patient Characteristics

Thirteen patients were included in this study (12 men, 1 woman). The average patient age at diagnosis was 13.2 ± 4.8 years. The patients had received diagnoses of Ewing’s sarcoma, osteosarcoma, non-Hodgkin lymphoma, acute lymphocytic leukemia, Langerhans cell histiocytosis, neuroblastoma, acute myeloid leukemia, germ cell tumor, and synovial sarcoma (Table 1). Forty-one chemotherapy blocks were reviewed, of which 21 (51.2%) blocks were high emetogenic cases and 20 (48.8%) were moderate emetogenic cases (Table 1).

### 3.2. Olanzapine Usage

Twenty blocks (48.8%) used olanzapine for prophylaxis and 21 (51.2%) used olanzapine for rescue (Table 2). The duration of olanzapine administration was 3 ± 2 days at a dose of 0.07 ± 0.04 mg/kg/dose and 2.50 ± 1.37 mg/m^2^/dose. The median number of chemotherapy blocks that used olanzapine per patient was three (range, 1–16). Thirty-four (82.9%) blocks used once-daily dosing and seven (17.1%) used twice-daily dosing.

### 3.3. Efficacy of Olanzapine

In terms of overall CIV control, 31 (75.6%) blocks achieved a CR, 6 (14.6%) blocks achieved a PR, and 4 (9.8%) blocks showed NR. In 35 of 41 blocks (87.4%), no vomiting was recorded after olanzapine use. Two (4.9%) patients vomited once, and four (9.6%) patients vomited more than once after olanzapine administration.

**Figure 1:**
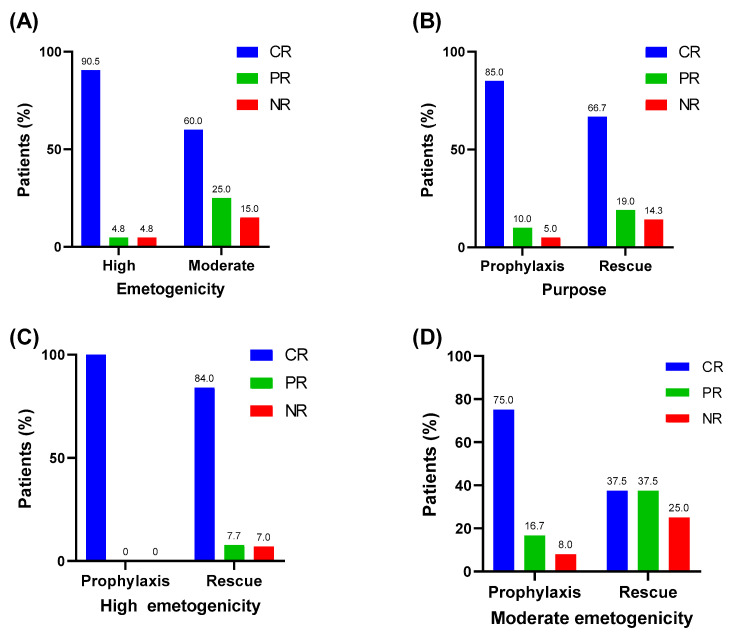
(**A**) Efficacy of olanzapine according to emetogenicity; (**B**) Efficacy of olanzapine according to purpose; (**C**) Efficacy of olanzapine according to purpose for high emetogenicity chemotherapy; (**D**) Efficacy of olanzapine according to purpose for moderate emetogenicity chemotherapy. CR, complete response; PR, partial response; NR, no response.

CR was achieved after olanzapine administration in 19 of 21 blocks (90.0%) of HEC; PR and NR were achieved for one block (4.8%) each. Of the 20 blocks of MEC, CR was achieved in 12 blocks (60%), PR in 5 blocks (25.0%), and NR in 3 blocks (15.0%) (Figure 1A).

CR was achieved in 17 of 20 blocks (85%) when olanzapine was administered for prophylactic purposes; PR and NR were achieved in two (10%) and one (5%) blocks, respectively. When administered for rescue use, CR was achieved in 14 blocks (66.7%), PR in 4 blocks (19.0%), and NR in 3 (14.3%) blocks (Figure 1B).

In HEC, all eight blocks showed a CR when olanzapine was used for prophylaxis. When olanzapine was administered as a rescue in HEC, 11 (84.4%) blocks showed CR, 1 block (7.7%) showed PR, and 1 block (7.7%) showed NR (Figure 1C). In MEC, nine (75%) blocks showed CR, two (16.7%) blocks showed PR, and one block (8.3%) showed NR when olanzapine was used as a prophylaxis. When olanzapine was used for a rescue purpose in MEC, three blocks (37.5%) showed CR, two blocks (37.5%) showed PR, and one block (25.0%) showed NR (Figure 1D).

If there was a side effect of sedation due to taking it once a day, it was administered in two divided doses. In addition, one dose was effective, but if nausea and vomiting occurred again in the evening, it was administered once more. The resulting effect was good, and sedation side effects were reduced.

### 3.4. Adverse Events during Olanzapine Treatment

The most common adverse effect of patients was somnolence, and symptoms occurred in eight (19.5%) blocks. Hyperglycemia occurred in six blocks (14.6%) and fatigue and disturbed sleep occurred in one block (2.4%) each.

## 4. Discussion

Despite the appropriate use of prophylactic antiemetics, breakthrough and refractory CIV often occurs, and there is a lack of information on its effectiveness in children. We determined the efficacy and safety of olanzapine for CIV control in 13 children undergoing 41 chemotherapy blocks at a single institution. When olanzapine was administered along with standard AEDs for CIV prophylaxis and rescue, 85% and 66.7% of children achieved complete CIV control, respectively. In addition, associated adverse events reported with its use were transient and clinically insignificant.

Flank et al. conducted a retrospective analysis of children <18 years old who were administered olanzapine for acute CINV control. In their study, the CR rate during HEC prophylaxis was 68%. The olanzapine dose was only associated with a sedative effect but not with CIV control. The most reported adverse events were sedation (7%) and increased transaminase levels (20%) [3]. The mean initial single olanzapine dose administered was 0.10 ± 0.051 mg/kg/dose, and the maximum single dose was 10 mg. In our study, CR rates of HEC prophylaxis and rescue were 100% and 84.0%, respectively, and the most frequent adverse event was somnolence (15.6%). The mean olanzapine dose was 0.07 ± 0.04 mg/kg/dose (2.50 ± 1.37 mg/m^2^/dose).

Flank et al. analyzed the initial dose of olanzapine in their study; in contrast, we analyzed the final dose, as dose modification was required to address sedation and unsatisfactory outcomes. The probable reason that the olanzapine dose was less than that reported by Flank et al. is that the dose was reduced owing to somnolence. Despite the low olanzapine dose, there may have been a racial difference in drug susceptibility, lessening the effect of antiemetics.

Venkatraman et al. conducted randomized phase III trial comparing olanzapine and metoclopramide for breakthrough CIV. CR rates were significantly higher in the olanzapine arm compared with the metoclopramide arm for nausea and vomiting. Hyperglycemia and drowsiness were more commonly seen in the olanzapine arm [16].

Some prospective trials have focused on olanzapine for CINV control in adult patients with cancer. Navari et al. conducted a randomized phase III trial to compare olanzapine and metoclopramide for breakthrough CINV in adult patients undergoing HEC. They found that olanzapine was better at controlling breakthrough emesis and nausea than metoclopramide [12]. In 2011, Navari et al. compared the effectiveness of olanzapine and aprepitant in adult patients undergoing HEC. Patients were randomly assigned to be administered olanzapine, palonosetron, dexamethasone (OPD) or aprepitant, palonosetron, and dexamethasone regimens. The CR rate was similar in both groups, but nausea control was better with the OPD regimen. Furthermore, the OPD regimen was more effective than the aprepitant, palonosetron, and dexamethasone regimen in controlling acute and delayed CINV [21].

Jeon et al. conducted a randomized, double-blind, placebo-controlled study focusing on whether olanzapine can reduce CINV in adult patients receiving MEC in Korea. Although they observed similar CR rates in the olanzapine and placebo groups, nausea control was better in the olanzapine group. In addition, they found that an OPD regimen significantly improved the quality of life of patients and reduced the frequency of vomiting [9].

In contrast to adults, there have been few prospective trials to evaluate olanzapine for CINV prevention in children. Flank et al. evaluated the feasibility of a trial assessing how olanzapine may contribute to CINV control in pediatric oncology patients. They enrolled 15 children who were administered at least half of the planned olanzapine dose (0.14 mg/kg/dose; maximum 10 mg/dose). Vomiting was completely controlled in eight patients in both the acute and delayed phases but 14 patients experienced nausea. They concluded that a pediatric trial of olanzapine for CINV control is feasible [2].

The limitation of this study is that the first is that it is a retrospective study involving a small number of patients, and this is considered a major limitation in drawing a clear conclusion. Second, the group of patients included in the study were patients with heterogenous characteristics, and the diagnosis was varied. Considering the differences between disease and its treatment, this is also a limitation of this study.

In this study, we retrospectively analyzed pediatric patients who were administered olanzapine for antiemetic purposes over a 26-month period at a single center. Olanzapine showed excellent efficacy in controlling CIV in pediatric patients who received MEC and HEC regimens. As this was a retrospective study, the analysis of nausea was insufficient. Nevertheless, this is the first study to demonstrate the efficacy of olanzapine for CINV control in Asian children. Our findings will contribute to the approval of olanzapine by the Korea Food and Drug Administration and national health insurance coverage for the treatment of and prophylaxis against CINV in pediatric patients with cancer in Korea and other countries.

## 5. Conclusions

Olanzapine, in combination with standard AEDs, showed promising results for both prophylaxis and rescue therapy of CINV in children. Furthermore, it showed few adverse effects, other than sedation, and was safe in children, which has long been recognized by psychiatrists. Olanzapine may be an important option for effectively controlling CINV in pediatric patients with cancer and subsequently improving their quality of life. There are limitations in reaching conclusions with small retrospective studies. Therefore, a prospective multicenter controlled study is needed to confirm these findings.

## Figures and Tables

**Table 1:** Baseline characteristics of patients.

	Characteristics	*N* (%)
Age	Mean ± SD	13.2 ± 4.8
	Median (range)	15.1 (4.0–18.0)
Sex	Male	12 (92.3)
	Female	1 (7.7)
Diagnosis	Ewing’s sarcoma	3 (23.1)
	Acute lymphoblastic leukemia	2 (15.4)
	Acute myeloid leukemia	2 (15.4)
	Langerhans cell histiocytosis	1 (7.7)
	Neuroblastoma	1 (7.7)
	Synovial sarcoma	1 (7.7)
	Osteosarcoma	1 (7.7)
	Non-Hodgkin lymphoma	1 (7.7)

**Table 2:** Olanzapine use during chemotherapy blocks.

Characteristic		*N* (%)
Chemotherapy emetogenicity	High	21 (51.2)
	Moderate	20 (48.8)
Reason for olanzapine use	Prophylaxis	20 (48.8)
	Rescue	21 (51.2)
Chemotherapy block (days)		4 ± 2
Duration of olanzapine use (days)		3 ± 2
Olanzapine dose	mg/kg/dose	0.07 ± 0.04
	mg/m^2^/dose	2.50 ± 1.37
Olanzapine frequency	Once daily	34 (82.9)
	Twice daily	7 (17.1)
